# Genetic Structure of Racing Pigeons (*Columba livia*) Kept in Poland Based on Microsatellite Markers

**DOI:** 10.3390/genes13071175

**Published:** 2022-06-29

**Authors:** Angelika Podbielska, Anna Radko

**Affiliations:** Department of Animal Molecular Biology, National Research Institute of Animal Production, Krakowska 1, 32-083 Balice, Poland; anna.radko@iz.edu.pl

**Keywords:** homing pigeons, racing, genetic diversity, population structure, individual identification, parentage testing, gene flow, microsatellite markers

## Abstract

Pigeons played a major role in communication before the invention of the telephone and the telegraph, as well as in wars, where they were used to carry information and orders over long distances. Currently, numerous sports competitions and races are held with their participation, and their breeding is demanding not only for breeders, but also for the birds themselves. Therefore, an analysis of the genetic structure of racing pigeons kept in Poland was undertaken on the basis of 16 microsatellite markers, as well as the evaluation of the microsatellite panel recommended by ISAG. For this purpose, Bayesian clustering, a dendrogram, and Principal Coordinate Analysis were conducted. In addition, statistical analysis was performed. Based on this research, it was observed that racing pigeons are genetically mixed, regardless of their place of origin. Moreover, genetic diversity was estimated at a relatively satisfactory level (Ho = 0.623, He = 0.684), and no alarmingly high inbreeding coefficient was observed (F = 0.088). Moreover, it was found that the panel recommended by ISAG can be successfully used in Poland for individual identification and parentage testing (PIC = 0.639, CE-1P = 0.9987233, CE-2P = 0.9999872, CE-PP = 0.99999999).

## 1. Introduction

Pigeons (*Columba livia*) are popular all over the world [1]. They are mainly bred for meat, as ornamental birds, and as racing birds [2]; their enthusiasts and hobbyists number in the thousands, with breeders all around the world [1]. There are also many feral pigeons, which are a common human commensal found in cities [3].

Pigeons were most likely domesticated 5000 years ago [2], and, to date, there are an estimated 350 breeds of domestic pigeons [4], whose ancestor was the rock dove [2,5]. It is estimated that their domestication took place in the Eastern Mediterranean [5,6], and it was most likely not a deliberate human act but rather occurred on a commensal pathway [6]. Current breeds of pigeons are extremely diverse in terms of their traits due to numerous selection processes [1]; therefore, it is possible that the domestication of the pigeon took place in different places at different times [5,7]. It cannot be ruled out that domestication may have occurred even before the Neolithic era [5]. There is still considerable uncertainty regarding the domestication of pigeons [6].

Ancient societies used pigeons to carry messages and as meat, and pigeon waste was used as fertilizer [2]. Pigeons also played an important role in culture and art [5]. In Europe, the crusaders introduced knowledge about breeding and the high utility of pigeons; however, in the Middle Ages, the use of these birds was a privilege of the nobility [8]. Because of their ability to return to the loft even from considerable distances and their excellent spatial orientation, pigeons have played a significant role in human civilization as information carriers [9]. During both World Wars, pigeons were used to convey information and orders. The most famous pigeon from World War I was ‘Cher Ami’, serving in the United States Army, which, despite her injuries, saved 194 soldiers, for which she was awarded a *Cross of War* [8]. Interestingly, documents from the Ukrainian archives, regarding the administrative prohibition of breeding homing pigeons in Poland, confirm that for the German occupation of World War I, the possession of these birds in private hands was considered a threat to the security of the lands occupied by the German Reich [8]. Civil pigeon breeding farms were shut down for fear they could be used by Russian agents to transmit orders and information. The owners who did not follow the decision were brought to court and were threatened with a fine or even imprisonment [8].

Currently, homing pigeons no longer carry letters, they only take part in competitions. For many years, pigeon racing has enjoyed great interest across the world as well as in Poland. This is a type of sport that involves the release of homing pigeons at various distances, which then return to their starting point. The time it takes an animal to travel a certain distance is measured, and the bird’s flight rate is calculated and compared to all other pigeons in the race to determine which animal returned with the fastest speed [10,11,12].

Racing pigeons are also called Racing Homer or homing pigeons [13] and they originated in Belgium, where in the middle of the 19th century a representative of this breed was created as a result of the continuous crossing of several breeds of pigeons [2]. Racing pigeons can travel up to 1000 km per day and reach speeds of over 100 km/h, with an average of 60 km/h. These abilities are not possessed by other breeds of pigeons [9]. With the potential to achieve better and better results in competitions, the prices of birds are rising [10]. The record price recently was €1.6 m for a Belgian ‘New Kim’ female sold in 2020 to a breeder from China [14].

In post-war times, racing pigeons in Poland were bred mainly by workers, but later this activity became popular among representatives of other professions, including doctors, teachers, and even priests. Organized pigeon competitions during the summer period were held almost every Sunday and enjoyed the interest of the local population, even those who did not breed pigeons. Currently, pigeon breeding in Poland is usually a family tradition, and the love for these birds passes from father to son [15]. Interestingly, pigeons bred in Poland are also successful in the international arena. The first place in the Federation Colombophile Internationale (FCI) competition, World’s Best Pigeons 2021, in the long distance category was won by ‘Eliud’, from Polish breeding [16,17]. Currently, The Polish Association of Racing Pigeon Breeders operates in Poland, with over 40,000 breeders and supporters, which was founded in 1926 [18]. This organization is also a member of the (FCI) [19].

The aim of the study was to investigate the population structure and genetic diversity of racing pigeons kept in Poland, as well as to examine the usefulness of microsatellite markers recommended by the International Society for Animal Genetics (ISAG) for the individual identification and parentage testing of these birds.

## 2. Materials and Methods

Biological material from feathers was collected from 519 racing pigeons. Some pigeons hatched in Poland, while some were purchased at various European auctions. It is also common for pigeon breeders from different countries to exchange birds with each other. This usually applies to the offspring, i.e., young pigeons whose parents were good players. For these reasons, the population of pigeons kept in Poland is variable and diverse. In this way, the samples were divided according to the identification data on the birds’ rings: Poland (PL; 364 samples), Belgium (BE; 91 samples), Germany (DV; 28 samples), Slovakia (SK; 17 samples), and the Netherlands (NL; 19 samples).

DNA was extracted with the Sherlock AX Kit (A&A Biotechnology, Gdynia, Poland) following the manufacturer’s suggested protocol. DNA concentration and quality were assessed using a NanoDrop (Thermo Fisher Scientific, Waltham, MA, USA).

Genomic DNA was amplified using the twelve microsatellite markers recommended by the ISAG as a core panel, four microsatellite markers as additional markers, and one marker, CHD, to determine the sex of each pigeon. The sex marker is not required by the ISAG (Table 1).

The reaction mixture contained 11.2 μL of Type-it Microsatellite PCR Kit (QIAGEN GmbH, Hilden, Germany), 1.2 μL of primer mix, and 1 μL of DNA (10 ng/μL). The PCR conditions for all reactions consisted of an initial denaturation of 95 °C for 5 min, followed by 35 cycles of 95 °C for 1 min, 55 °C for 1 min, and 72 °C for 1 min, with a final extension step of 72 °C for 30 min. Capillary electrophoresis was performed using a 3130xl Genetic Analyser (Applied Biosystems, Foster City, CA, USA) and contained 11 μL of formamide, 0.4 μL of GeneScan™ 500 LIZ™ dye Size Standard (Applied Biosystems), and 1 μL of PCR product. Samples were denatured for 5 min at 95 °C. The electrophoresis results were analysed using GeneMapper v. 4.0 (Applied Biosystems).

Pairwise F_ST_ (parameter of population differentiation) and gene flow (Nm) values, number of different alleles (Na), number of effective alleles (Ne), Shannon’s Information Index (I), a deficit of heterozygotes (P), the Hardy–Weinberg equilibrium using the chi-square test (HWE), observed heterozygosity (Ho), expected heterozygosity (He), and inbreeding coefficient (F), as well as F-statistics parameters: the inbreeding coefficient within individuals relative to the subpopulation (Fis), the inbreeding coefficient within individuals relative to the total (Fit), and the inbreeding coefficient within subpopulations relative to the total (Fst), were calculated using GenAlEx 6.5 [20,21]. The polymorphism information content (PIC) and parentage testing parameters: the null allele frequencies (Fnull), non-exclusion first parent (NE-1P), non-exclusion second parent (NE-2P), non-exclusion parent pair (NE-PP), non-exclusion identity (NE-I), non-exclusion sibling (NE-SI), combined exclusion probability-first parent (CE-1P), combined exclusion probability-second parent (CE-2P), and combined exclusion probability-parent pair (CE-PP), were calculated by CERVUS 3.0.7 [22].

Principal coordinate analysis (PCoA) was performed using GenAlEx [20,21]. A Nei genetic distance dendrogram [23] was constructed using the unweighted pair group method with arithmetic mean (UPGMA) [24]. The tree was visualised by The Interactive Tree Of Life iTOL v6 [25]. An analysis in STRUCTURE software 2.3.4 [26] was performed to determine the population structure. The analysis was performed with a length of Burnin period of 100,000 and 200,000 MCMC repetitions after Burnin. Set *K* ranged from 1 to 10, with 10 iterations for each *K*. The analysis was performed in two ways. In the first one, all pigeons were used as representatives of one specific breed (racing pigeons). In contrast, in the second, pigeons were divided into five groups according to the country of origin (PL, BE, DV, NL, SK). A STRUCTURE HARVESTER [27] was used to select the best *K* using Evanno’s model [28], and CLUMPAK was used for the summation and graphical representation of the obtained results [29].

## 3. Results

### 3.1. Genetic Diversity and Parentage Testing

The pigeons kept in Poland showed a relatively high level of genetic diversity; a total of 146 different alleles were observed. The average number of alleles per locus was 9.125, ranging from 4 alleles in CliμD19 to 19 alleles in PIGN12 (Table 2). Higher Ho than He was observed in the PIGN57 and PIGN10 markers. The highest F index was recorded for the PIGN12, while the lowest was for PIGN57. For the PIGN26 marker, the highest values of Ne, I, Ho, and He were recorded, and the lowest values for these parameters were recorded for CliμD35. The mean PIC was estimated at a satisfactory level of 0.639; however, extremely low values for the CliμD35 (0.275) marker and extremely high values for the PIGN26 (0.903) marker were noted.

Analogous results were obtained for the parameters strictly related to the analysis of parentage (NE–1P, NE–2P, NE–PP, NE–I, and NE–SI) (Table 3). In addition, the values that allowed the estimation of the probability of exclusion of an offspring after one or the other parent or after the parental pair were estimated at the level of 99.87%, 99.99%, and 99.999999%. A high frequency of null alleles was reported for the marker PIGN12 and CliμD19, while the lowest was for PIGN57.

F-statistics indicators (Table 4) for the entire study population differed depending on the marker. The lowest values of Fis and Fit were obtained for CliμT02, while the highest value of these indicators was estimated for PIGN12. In CliμD16, the highest level of gene flow and the lowest Fst were observed, compared with the opposite situation for CliμT17. The average value of Nm was as high as 9.775, which was a relatively high result. It was also observed that this value did not drop below 1 for any of the markers.

### 3.2. Population Structure

**Figure 1:**
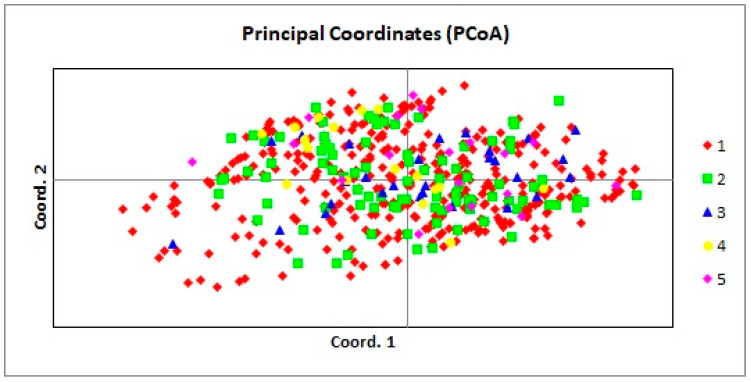
Principal coordinate analysis (PCoA) of all pigeons. 1—PL; 2—BE; 3—DV; 4—SK; 5—NL. Percentage of variation explained by the first 3 axes: 3.34, 2.89, and 2.45.

PCoA was performed on the complete dataset of 519 homing pigeon genotypes (Figure 1) in order to graphically present the relationship between individuals and groups of pigeons and to determine whether the division into these groups is supported by genetic variation.

A high level of overlapping was shown in the PCoA scatter plot of the analysis of the entire dataset. The first principal coordinate accounted for 3.34% of the total variation, while the second coordinate accounted for 2.89% of the total variation. There was no clear separation of individuals according to their origin.

**Figure 2:**
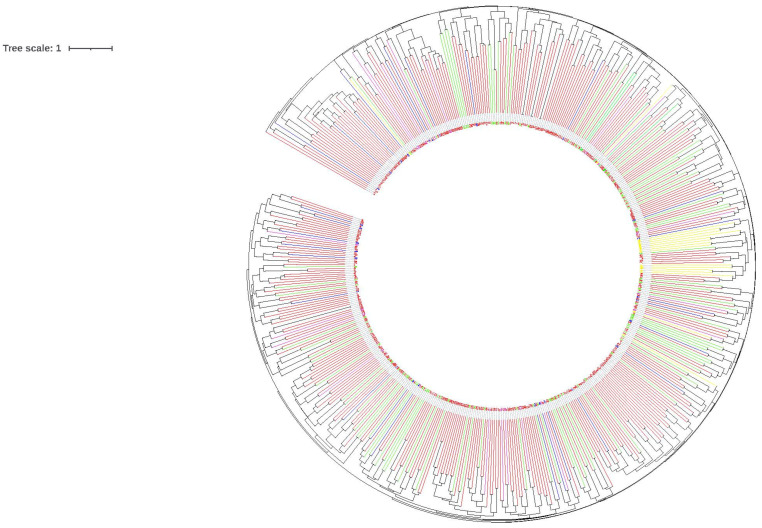
Dendrogram of genetic distance between all individuals with the UPGMA algorithm. Red—PL; green—BE; blue—DV; yellow—SK; pink—NL.

The obtained dendrogram did not reveal any separate genetic clusters related to the origin of the pigeons (Figure 2), confirming the genetically mixed nature of racing pigeons.

**Figure 3:**
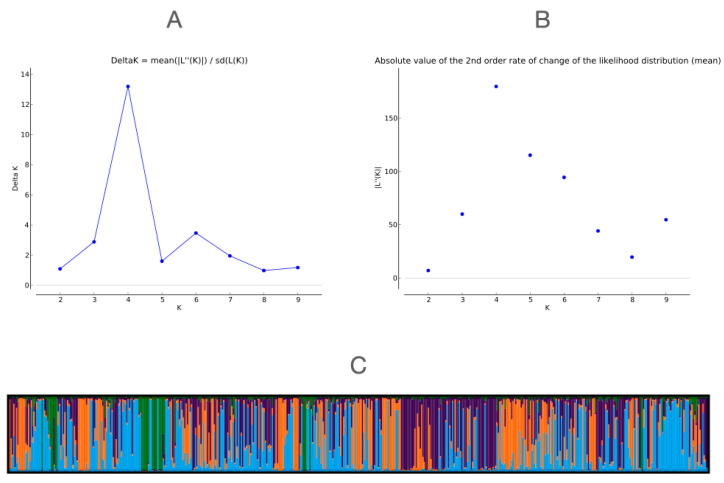
(**A**) Delta K values obtained with STRUCTURE HARVESTER. (**B**) Rate of change in the likelihood distribution (mean) obtained with STRUCTURE HARVESTER. (**C**) STRUCTURE software clustering at *K* = 4 on a dataset containing 519 individuals.

The Bayesian approach revealed that the most likely genetic structure for pigeons kept in Poland is a structure composed of four major genetic clusters (the best *K* = 4) (Figure 3). In this case, ten runs delivered an identical score (similarity score) of 0.991. These clusters, however, were not in line with the country of origin of the pigeons (Figure S3). The same results were obtained when the pigeons were analyzed as one group (1 breed—racing pigeons) and when they were divided into five groups according to the country of origin.

Additional information from STRUCTURE software analysis (Tables S1 and S2, Figures S1 and S2), pairwise F_ST_ values (Table S3), and gene flow values between populations (Table S4) are included in the Supplementary Materials.

## 4. Discussion

### 4.1. Genetic Diversity

As is well-known, the ancestors of modern racing pigeons carried information during the course of the two World Wars and, indeed, experienced a decline in genetic diversity. Then, poverty and hunger decimated the farms of that time and disrupted the free interbreeding. Due to this phenomenon and the inbreeding procedures to accumulate flight predispositions, these birds were significantly exposed to the loss of genetic diversity.

So far, the genetic diversity of Polish meat pigeons with the participation of microsatellite DNA [30], and fancy breeds with the participation of mitochondrial DNA [31] has been examined, and, in both cases, it was relatively low.

In our analysis, the number of alleles per locus varied from 4 to 19, while in feral pigeons, using only 7 markers, these numbers fluctuated between 9 and 26 [32], which is a much higher genetic richness. This is understandable as wild pigeons are free-living animals that can travel independently and have better opportunities to find an unrelated breeding partner. In turn, the average value of the number of different and effective alleles was estimated at 9.125 and 4.046, respectively, which were much higher results than those obtained from Italian pigeons with 4.3 and 2.7 [33], or Egyptian pigeons with 9.091 and 2.575 [34]. A higher mean effective number of alleles indicates that the population can retain the original gene and avoid new changes under the pressure of genetic drift and artificial selection.

In our research, we obtained higher mean values of Ho and He than in the population of Egypt [2]. These results show that the genetic diversity of pigeons kept in Poland is not endangered; however, breeding steps could be taken to try to increase it. Unfortunately, this is a fairly common phenomenon, as other researchers have obtained even lower rates of these parameters. On the other hand, in our study, we obtained lower values of expected and observed heterozygosity compared to the studies with the Scaly-naped Pigeon, an endemic species, which due to the limited range of habitats and smaller population sizes, is characterized by less genetic diversity and a greater risk of inbreeding than continental species [35]. In turn, extremely low genetic diversity was obtained for the Red-headed Wood Pigeon, which is an endemic endangered species that has also undergone a bottleneck [36].

In turn, genetic variability estimated using the Shannon’s Information Index was observed at a higher level than in pigeons tested in China [37] or in Spain [38], but its values in the various markers were not similar, but, rather, in contrast. These results again indicate a high level of breeding selection, which disrupted the environmental equilibrium causing instability in the population. In our research, 9 of the 16 markers noted a significant deviation from the HWE, but in the Egyptian breeds, 4 of the 11 markers showed a similar result [34]. In the case of our work, the deviations from the HWE confirm that there is no random mating among racing pigeons, but on the contrary, mating by kinship. In addition, the frequent presence of null alleles also adversely affects the HWE (PIGN12, CliμD19). Moreover, all markers except PIGN10 and PIGN57 indicated lower Ho than He values. This can be explained by the so-called founder effect, where the genetic pool narrows down and grows over time, but only based on the pool founding. Egyptian pigeons [2,34] had significantly lower levels of inbreeding coefficients (F) than ours. The average coefficient of inbreeding estimated by us at the level of 0.088 was also much lower than that of most meat pigeons that are artificially selected for breeding pairs [30]. F values range from −1 to 1. Inbreeding coefficient values > 0 indicate an excess of homozygotes in the population, which may indicate an inbred population. The inbreeding effect may be due to breeding selection, genetic drift, or a bottleneck effect. The result we obtained definitely indicates the lack of random crossings in breeding pigeons. This result also indicates an intensive selection among the racing pigeon populations. However, in the case of racing pigeons kept in Poland, the level of inbreeding seems to be at a controlled level, although Polish breeders admit that they use inbreeding in order to obtain birds that could have outstanding speed flying abilities. This selection is often carried out on the basis of genotypes obtained as a result of the study of the polymorphism of the LDHA [39,40] and DRD4 [41] genes, which are very popular among breeders not only in Poland.

The genetic differentiation obtained in our work with the use of F-statistic indices and gene flow across 16 microsatellite markers was clearly smaller than in the case of Egyptian [2,34] and Italian [33] pigeons. This proves that there is little differentiation between pigeon populations kept in Poland, which at the same time means a large gene flow, the average value of which was revealed at the level of 9.775. The movement of organisms causes the flow of genes and, hence, greater maintenance of genetic diversity and the ability to adapt. Reduced genetic diversity and increased self-rearing may result in less effective reproduction or even reduced survival [3]. Nevertheless, it should also be noted that the mean of all fixation indices was positive, which may be a consequence of breeding procedures consisting in mating with relatives in order to obtain offspring with predisposition to flights. The positive values of these parameters strictly indicated a deficit of heterozygotes among the studied birds. However, studies on French feral pigeons [42] have shown that mating with genetically similar mates can have adaptive benefits, a phenomenon that is much more common than previously thought. Despite the adverse effects of inbreeding on offspring, it increases the parent’s inclusive fitness, an individual that mates with a relative will help that relative spread identical genes by origin [42].

### 4.2. Individual Identification and Parentage Testing

The current microsatellite panel recommended by ISAG for pigeons was proposed by the Committee of the Society, which is now called ‘Applied Genetics and Genomics in other Species of Economic Interest’ and has been successfully used by other standardized laboratories that also provide services in the field of personal identification and parentage testing. VHL Genetics undertakes pigeon population studies with the use of these markers [43]. The authors in this study, based on the statistical analysis, demonstrated the usefulness of this panel, paying attention to parameters such as combined exclusion probability-first parent, combined exclusion probability-second parent, and combined exclusion probability-parent pair, where they obtained the values of 99.86%, 99.99%, and 99.99%, respectively. We obtained almost identical values in our research, which confirms that the panel recommended by ISAG for pigeons is useful and can be successfully used for the individual identification and parentage testing of these birds in different countries. The values obtained in our study were higher and more satisfactory than those obtained on other markers used for traceability in Taiwan [44].

When it comes to the parameters strictly used for the parentage testing in our study, the PIGN26 marker was the highest. Values indicating its high potential for parental analysis were estimated. The CliμD35 marker was the lowest. Interestingly, previous studies showed the same results [43]. Therefore, the replacement of the CliμD35 marker with another marker may be considered in the future in the ISAG panel. 

Interestingly, despite the similarity of results between our laboratory and VHL Genetics [43], the exception in this case was the F (null) parameter. In our results, the marker PIGN12 showed the highest value, while in the Netherlands, the highest value was estimated for CliμD19. However, it should be noted that the marker CliμD19 also showed a high value in our research. Interestingly, in feral pigeons, a high degree of occurrence of the zero allele was observed in this marker [32,42]. In routine parentage testing in the case of bilateral tests involving the parental pair, we observed the phenomenon of the presence of two different homozygotes, one in the offspring and the second in one of the parents, in the PIGN12 marker. Not only that, the phenomenon is not as rare as it seems. It was observed much less frequently in the CliμD19 marker, which is consistent with the performed statistical analysis.

It should be noted that pigeon parentage testing in Poland is carried out in accordance with the ISAG recommendation. The nomenclature is standardized in ISAG Pigeon Comparison Tests (PCTs) and, therefore, comparable internationally with the results from other laboratories that also have standardization. The first proficiency PCT took place in 2013/2014 and to date, four such PCTs have already been carried out. The last one was organized in collaboration with ISAG and the National Research Institute of Animal Production [10].

The CHD marker given in this study was not included in the later analyses due to the fact that in pigeons it determines only two variants, the W allele and the Z allele, and is used to determine sex. This marker is also not recommended in the basic and additional ISAG panel. The analysis with its participation is important since in young pigeons it is often impossible to determine the sex from the external appearance. In pigeon breeding, at its early stages, it is important to know whether the bird is a female or a male; therefore, this analysis is very popular.

### 4.3. Genetic Structure

The population of racing pigeons kept in Poland is diverse, as breeders, not only from Poland but also from Europe, buy and sell birds at various auctions, and, sometimes, they even exchange them. The owners are still looking for the perfect birds with which they could achieve the highest results in sports competitions. However, this sport, as all others, is very demanding. The breeding of racing pigeons requires intensive selection and breeding work, training, feeding, and keeping the birds in proper condition. In addition, various sophisticated methods are often used to increase the incentives for the pigeons to return to the nest [9].

In our population, the Bayesian approach revealed four major genetic clusters unrelated to the pigeon’s country of origin. This can be explained by the fact that Belgium is the creator of the modern racing pigeon, and it was in this country that the breeding of these birds began; then, the knowledge and passion spread to other European countries and beyond. The four genetic clusters likely form the descendants of the first genetic lines of pigeons that were selected and then crossed with relatives, as a result of which a new breed of pigeons was created. However, this hypothesis should be confirmed by examining more samples with racing pigeons from other countries. Nevertheless, the topic is worth further research. Interestingly, there were also four main genetic clusters obtained in the wild pigeon group tested in Italy [45]. Not only that, a relationship has been noted between domestic and feral pigeons. It is largely assumed that feral pigeons are derived from domestic breeds [45], which may explain the observed genetic structure. Moreover, it is certain that some birds do not come back from longer flights. It is estimated that up to 20% of the birds that start the race do not return to the starting point [1]. Some of them fall victim to predators, some suffer a collision with architectural buildings, and some lose their way home due to disorientation and start living in the wild.

The PCoA of all individuals, revealed the genetically mixed nature of pigeons tested by us. The analysis did not confirm that the pigeons were grouped according to their country of origin of the birds. There was a slight variation in genotypes, indicating a high level of admixture between individuals. Most likely, this can be explained by the fact that all racing pigeons were created in one country, and were bred for one purpose—to quickly find a way home over often very long distances, while the next step was their distribution spread worldwide. Genetic relatedness between racing pigeons was also demonstrated through genome sequencing [46], where pigeons from various European and U.S. breeders were used. It was also found that racing pigeons are more genetically similar to each other than to other breeds, which, according to the authors, could be the result of the lack of crossing of racing pigeons with other breeds, due to the high competition in breeding selectively aimed at high flying efficiency. It is also worth mentioning here, that an analysis of the Italian racing pigeons also revealed a genetic link between the Italian racing pigeons and the English breed ‘Carrier’, which was once used as a message carrier [33]. Moreover, among the nine feral populations and the six Italian breeds, there was no clear separation between the two groups [45]. Wild pigeons are genetically similar to racing pigeons [45]. It is likely related to the aforementioned fact that some carrier pigeons get lost during races, and some begin to live in the wild.

UPGMA analysis indicated no obvious segregation of genetic profiles among all pigeons, which confirms the genetically mixed nature of the population, as also demonstrated by the PCoA analysis. Inconsistencies between the phylogenetic clades of different species of pigeons were also noticed in the analysis of mitochondrial DNA, analysed to determine the genetic structure of these birds and to study the universality of genetic primers [47]. The authors indicated the possibility of birds migrating as the reason. Interestingly, the trees generated from the microsatellite data of wild pigeons tested in Italy, where the feral and domestic groups were differently related to each other, did not show any significant structure [45].

## 5. Conclusions

This paper provided a detailed analysis of the population structure and genetic diversity of racing pigeons kept in Poland. Research revealed that their genetic structure is mixed; the pigeons did not differentiate into groups depending on their original place of origin. The genetic diversity of pigeons kept in Poland was found to be at a relatively satisfactory level. Despite the specific breeding procedures used, no alarming values were recorded for the inbred index. In addition, it was also found that the panel recommended by ISAG was useful for individual identification and parentage testing; however, in the future, ISAG could be used to replace low polymorphic markers and those with a tendency to null alleles with others. It also seems justified to monitor the genetic diversity of Polish pigeon populations in the future, due to the specific breeding procedures used for this group of animals.

## Figures and Tables

**Table 1:** Characteristics of 17 microsatellite loci.

Locus	Forward	Reverse	Dye	Size Range (bp)	Primer Concentration F + R (μM)	Panel
CliμD11	CCAATCCCAAAGAGGATTAT	ACTGTCCTATGGCTGAAGTG	6-FAM	78–110	2.0	Core
CliμT43	GGGAAAGGAAATTTGACACTG	ACTGTCGATGCCATTAAGAC	6-FAM	191–229	1.0	
CliμD01	GATTTCTCAAGCTGTAGGACT	GTTTGATTTGGTTGGGCCATC	VIC	75–130	1.4	
PIGN57	CTCTTGTATGTCCATCTGAAC	ACCCATTTACCACTCTCTAA	VIC	153–189	1.8	
CliμT13	CTGTCGAGCAGTAACAGTCC	GTTTGCAAGCCCTGGTTATCTCA	VIC	198–240	2.0	
CliμD16	GCAGTGATAAAGTTCTGGAACA	GTTTGCCTCACCGTGACATCA	NED	75–185	2.0	
CliμD19	CTGCCCGTTTCTTCTAATGCAC	GTTTGGATTTCTGGGAGTGTATG	NED	186–204	1.8	
CliμT02	AGTTTTAATGAAGGCACCTCT	TGTAGCATGTCAGAAATTGG	PET	93–113	1.4	
CliμD17	TCTTACACACTCTCGACAAG	GTTTCCACCCAAATGAGCAAG	PET	116–130	1.2	
CliμD35	GGGAGCTTAAGGGATTATTG	ATTCCTTGCATGCCTACTTA	PET	173–195	1.2	
CliμT17	ATGGGTTTGGAGATGTTTTG	GTTTGATGGAGTTGCTATTTTGCT	PET	209–259	2.0	
PIGN04	GGTTTTTCTGTTTCCTCACG	GGGATTCTGGGATTATTTTTTC	PET	273–327	0.4	
PIGN15	TTTCCTTTCATTTGCTGTGG	AACCAGGCATTGGAGTCTTT	6-FAM	126–154	2.4	Additional
PIGN10	TTCCACTGAATGGGTCTCAG	CTGCCAGAAGGTAAATGACAC	6-FAM	271–325	2.4	
PIGN26	TCACTGTATTCACCAAAGTCTG	CAATGTGGGGGCGTCTATG	VIC	364–494	0.6	
PIGN12	CAGATCCAGCAGTCTTGAAG	CCCATCTAATGCGATAAATCC	NED	241–371	4.0	
CHD	CTCCCAAGGATGAGRAAYTG	ATGGAGTCACTATCAGAT	VIC	266–290	1.0	Without ISAG recommendation

**Table 2:** Genetic diversity parameters across 17 microsatellite markers.

Locus	Na	Ne	I	P	HWE	Ho	He	PIC	F
CliμD11	8	3.458	1.457	0.000	***	0.674	0.711	0.670	0.051
CliμT43	8	4.835	1.687	0.117	ns	0.751	0.793	0.764	0.053
CliμD01	12	5.247	1.880	0.000	***	0.794	0.809	0.784	0.019
PIGN57	8	3.397	1.384	0.000	***	0.711	0.706	0.655	−0.008
CliμT13	7	4.267	1.538	0.888	ns	0.750	0.766	0.727	0.021
CliμD16	11	3.401	1.565	0.421	ns	0.697	0.706	0.674	0.012
CliμD19	4	2.016	0.723	0.000	***	0.374	0.504	0.382	0.258
CliμT02	6	2.137	0.882	0.000	***	0.493	0.532	0.431	0.073
CliμD17	6	2.520	1.131	0.002	**	0.572	0.603	0.538	0.051
CliμD35	6	1.456	0.562	0.243	ns	0.281	0.313	0.275	0.101
CliμT17	11	2.793	1.447	0.057	ns	0.588	0.642	0.617	0.085
PIGN04	7	2.542	1.224	0.000	***	0.549	0.607	0.552	0.095
PIGN15	6	3.251	1.333	0.601	ns	0.674	0.692	0.645	0.026
PIGN10	10	4.399	1.653	0.705	ns	0.775	0.773	0.740	−0.002
PIGN26	17	11.173	2.514	0.040	*	0.884	0.910	0.903	0.029
PIGN12	19	7.849	2.324	0.000	***	0.399	0.873	0.860	0.543
Mean	9.125	4.046	1.457			0.623	0.684	0.639	0.088

Key: ns = not significant, * *p* < 0.05, ** *p* < 0.01, *** *p* < 0.001; Na, number of different alleles; Ne, number of effective alleles; I, Shannon’s Information Index; P, a deficit of heterozygotes; HWE, Hardy–Weinberg equilibrium; Ho, observed heterozygosity; He, expected heterozygosity; PIC, polymorphic information content; F, inbreeding coefficient.

**Table 3:** Parentage testing indices across 17 microsatellite markers.

Locus	NE–1P	NE–2P	NE–PP	NE–I	NE–SI	F(Null)
CliμD11	0.695	0.517	0.327	0.124	0.426	0.025
CliμT43	0.582	0.403	0.220	0.072	0.371	0.026
CliμD01	0.544	0.369	0.187	0.061	0.361	0.008
PIGN57	0.712	0.542	0.363	0.138	0.432	−0.007
CliμT13	0.636	0.457	0.277	0.094	0.391	0.010
CliμD16	0.685	0.502	0.299	0.118	0.427	0.008
CliμD19	0.873	0.806	0.709	0.368	0.590	0.149
CliμT02	0.857	0.760	0.638	0.320	0.564	0.038
CliμD17	0.804	0.657	0.492	0.223	0.504	0.029
CliμD35	0.951	0.856	0.760	0.510	0.721	0.055
CliμT17	0.744	0.556	0.345	0.153	0.467	0.045
PIGN04	0.794	0.636	0.458	0.209	0.499	0.055
PIGN15	0.725	0.552	0.370	0.142	0.439	0.015
PIGN10	0.612	0.433	0.246	0.084	0.385	−0.001
PIGN26	0.309	0.182	0.054	0.015	0.299	0.015
PIGN12	0.404	0.252	0.095	0.029	0.321	0.373
Mean	0.683	0.530	0.365	0.166	0.450	0.053

CE–1P = 0.9987233; CE–2P = 0.9999872; CE–PP = 0.9999999; NE–1P, non-exclusion probability first parent; NE–2P, non-exclusion probability second parent; NE–PP, non-exclusion probability parent pair; NE–I, non-exclusion probability identity; NE–SI, non-exclusion probability sibling; F(null), estimated frequency of null allele; CE–1P, combined exclusion probability-first parent; CE–2P, combined exclusion probability-second parent; CE–PP, combined exclusion probability-parent pair.

**Table 4:** F-statistics and estimates of Nm coefficients across 17 microsatellite markers.

Locus	Fis	Fit	Fst	Nm
CliμD11	0.056	0.094	0.041	5.894
CliμT43	0.047	0.091	0.047	5.111
CliμD01	0.025	0.045	0.021	11.757
PIGN57	−0.024	0.030	0.053	4.502
CliμT13	−0.016	0.001	0.017	14.584
CliμD16	0.016	0.030	0.015	16.679
CliμD19	0.122	0.144	0.025	9.688
CliμT02	−0.092	−0.060	0.029	8.478
CliμD17	0.069	0.093	0.026	9.485
CliμD35	0.079	0.099	0.021	11.494
CliμT17	0.067	0.116	0.053	4.486
PIGN04	0.067	0.090	0.025	9.724
PIGN15	0.029	0.046	0.018	13.965
PIGN10	−0.025	−0.005	0.020	12.279
PIGN26	0.005	0.024	0.018	13.444
PIGN12	0.524	0.547	0.049	4.837
Mean	0.059	0.087	0.030	9.775

Fis, the inbreeding coefficient within individuals relative to the subpopulation; Fit, the inbreeding coefficient within individuals relative to the total; Fst, the inbreeding coefficient within subpopulations relative to the total; Nm, gene flow.

## Data Availability

Not applicable.

## Supplementary Materials

The following supporting information can be downloaded at: https://www.mdpi.com/article/10.3390/genes13071175/s1, Table S1: The Evanno table output from STRUCTURE HARVESTER on a dataset containing 519 individuals; Table S2: The Evanno table output from STRUCTURE HARVESTER on a dataset containing 519 individuals divided into five populations by country of origin; Table S3: Pairwise F_ST_ values, where: 1–PL; 2–BE; 3–DV; 4–SK; and 5–NL; Table S4: Pairwise population Nm values based on F_ST_ values, where: 1–PL; 2–BE; 3–DV; 4–SK; and 5–NL; Figure S1: Plot of mean likelihood L(K) and variance per K value from STRUCTURE HARVESTER on a dataset containing 519 individuals; Figure S2: Plot of mean likelihood L(K) and variance per K value from STRUCTURE HARVESTER on a dataset containing 519 individuals divided into five populations by country of origin; Figure S3: (A) Delta K values obtained with STRUCTURE HARVESTER. (B) Rate of change in the likelihood distribution (mean) obtained with STRUCTURE HARVESTER. (C) STRUCTURE software clustering at K = 4, where: 1–PL; 2–BE; 3–DV; 4–SK; and 5–NL.

## References

[1] Stringham S.A., Mulroy E.E., Xing J., Record D., Guernsey M.W., Aldenhoven J.T., Osborne E.J., Shapiro M.D. (2012). Divergence, convergence, and the ancestry of feral populations in the domestic rock pigeon. Curr. Biol..

[2] Ramadan S., Abe H., Hayano A., Yamaura J., Onoda T., Miyake T., Inoue-Murayama M. (2011). Analysis of genetic diversity of Egyptian pigeon breeds. J. Poult. Sci..

[3] Carlen E., Munshi-South J. (2021). Widespread genetic connectivity of feral pigeons across the Northeastern megacity. Evol. Appl..

[4] Shapiro M.D., Kronenberg Z., Li C., Domyan E.T., Pan H., Campbell M., Tan H., Huff C.D., Hu H., Vickrey A.I. (2013). Genomic diversity and evolution of the head crest in the rock pigeon. Science.

[5] Od Dzikiego Gołębia Skalnego do ras Współcześnie Hodowanych—PDF Free Download. http://docplayer.pl/59099216-Od-dzikiego-golebia-skalnego-do-ras-wspolczesnie-hodowanych.html.

[6] Pacheco G., Van Grouw H., Shapiro M.D., Gilbert M.T.P., Vieira F.G., Storz J. (2020). Darwin’s Fancy Revised: An Updated Understanding of the Genomic Constitution of Pigeon Breeds. Genome Biol. Evol..

[7] Johnston R.F., Janiga M. (1995). Feral Pigeons.

[8] Roguski R. (2015). Administracyjne ograniczenia hodowli gołębi pocztowych na Kresach Drugiej Rzeczypospolitej w świetle dokumentów źródłowych. Rocz. Kresowy.

[9] Gugołek A., Jastrzębska A., Strychalski J. (2016). Wykorzystanie gołębi i innych gatunków ptaków w rekreacji człowieka. Wiadomości Zootech..

[10] Podbielska A., Ropka-Molik K., Stefaniuk-Szmukier M., Radko A. (2021). Udział Instytutu Zootechniki PIB w organizacji Międzynarodowego Porównawczego Testu Biegłości Profilowania STR Gołębi w latach 2020/2021. Wiadomości Zootech..

[11] Gáspárdy A. (2017). Connection among Body Measurements and Flying Speed of Racing Pigeon. Int. J. Agric. Sci. Food Technol..

[12] Kabir A., Hawkeswood T.J., Makhan D. (2020). Pigeon Flying in the World: A Brief Review. Calodema.

[13] Shao Y., Tian H.Y., Zhang J.J., Kharrati-Koopaee H., Guo X., Zhuang X.L., Li M.L., Nanaie H.A., Dehghani Tafti E., Shojaei B. (2020). Genomic and Phenotypic Analyses Reveal Mechanisms Underlying Homing Ability in Pigeon. Mol. Biol. Evol..

[14] New Kim: Racing Pigeon from Belgium Sold for Record €1.6m—BBC News. https://www.bbc.com/news/world-europe-54953594.

[15] Świtała-Trybek D. (2002). “Gołębiorze” na Górnym Śląsku Rozważania o Subkulturze Hodowców Gołębi. Studia Etnol. I Antropol..

[16] FCI Fédération Colombophile Internationale WORLD BEST PIGEONS 2021 Category: LONG DISTANCE FINAL RESULTS. https://pigeonsfci.org/wp-content/uploads/2022/02/WBP_Final_Long-distance.pdf.

[17] Strona Główna—Artur Zambrzycki, Hodowla Gołębi Pocztowych. http://www.golebiezambrzycki.pl/.

[18] Polski Związek Hodowców Gołębi Pocztowych—Oficjalny Serwis Zarządu Głównego PZHGP. https://pzhgp.pl/.

[19] Pigeonsfci|Federation Colombophile Internationale. https://pigeonsfci.org/.

[20] Peakall R., Smouse P.E. (2012). GenAlEx 6.5: Genetic analysis in Excel. Population genetic software for teaching and research--an update. Bioinformatics.

[21] Peakall R., Smouse P.E. (2006). genalex 6: Genetic analysis in Excel. Population genetic software for teaching and research. Mol. Ecol. Notes.

[22] Kalinowski S.T., Taper M.L., Marshall T.C. (2007). Revising how the computer program cervus accommodates genotyping error increases success in paternity assignment. Mol. Ecol..

[23] Nei M. (1972). Genetic Distance between Populations. Am. Nat..

[24] Garcia-Vallvé S., Palau J., Romeu A. (1999). Horizontal gene transfer in glycosyl hydrolases inferred from codon usage in *Escherichia coli* and *Bacillus subtilis*. Mol. Biol. Evol..

[25] Letunic I., Bork P. (2021). Interactive tree of life (iTOL) v5: An online tool for phylogenetic tree display and annotation. Nucleic Acids Res..

[26] Pritchard J.K., Stephens M., Donnelly P. (2000). Inference of population structure using multilocus genotype data. Genetics.

[27] Earl D.A., VonHoldt B.M. (2012). STRUCTURE HARVESTER: A website and program for visualizing STRUCTURE output and implementing the Evanno method. Conserv. Genet. Resour..

[28] Evanno G., Regnaut S., Goudet J. (2005). Detecting the number of clusters of individuals using the software STRUCTURE: A simulation study. Mol. Ecol..

[29] Kopelman N.M., Mayzel J., Jakobsson M., Rosenberg N.A., Mayrose I. (2015). Clumpak: A program for identifying clustering modes and packaging population structure inferences across K. Mol. Ecol. Resour..

[30] Biała A., Dybus A., Pawlina E., Proskura W.S. (2015). Genetic diversity in eight pure breeds and urban form of domestic pigeon (*Columba livia* var. *domestica*) based on seven microsatellite loci. J. Anim. Plant Sci..

[31] Stefaniuk-Szmukier M., Andres K., Piórkowska K., Ropka-Molik K. (2021). Low diversity of mitochondrial DNA in fancy pigeons (*Columba livia*) revealed by partial D-loop sequencing. Anim. Genet..

[32] Jacob G., Prévot-Julliard A.C., Baudry E. (2015). The geographic scale of genetic differentiation in the feral pigeon (*Columba livia*): Implications for management. Biol. Invasions.

[33] Bigi D., Mucci N., Mengoni C., Baldaccini E.N., Randi E. (2016). Genetic investigation of Italian domestic pigeons increases knowledge about the long-bred history of *Columba livia* (Aves: Columbidae). Ital. J. Zool..

[34] Ramadan S., Dawod A., El-Garhy O., Nowier A.M., Eltanany M., Inoue-Murayama M. (2018). Genetic characterization of 11 microsatellite loci in Egyptian pigeons (*Columba livia domestica*) and their cross-species amplification in other Columbidae populations. Vet. World.

[35] Cambrone C., Cézilly F., Wattier R., Eraud C., Bezault E. (2021). Levels of genetic differentiation and gene flow between four populations of the Scaly-naped Pigeon, *Patagioenas squamosa*: Implications for conservation. Stud. Neotrop. Fauna Environ..

[36] Ando H., Ogawa H., Kaneko S., Takano H., Seki S.I., Suzuki H., Horikoshi K., Isagi Y. (2014). Genetic structure of the critically endangered Red-headed Wood Pigeon *Columba janthina nitens* and its implications for the management of threatened island populations. Ibis.

[37] Zhang X., He Y., Zhang W., Wang Y., Liu X., Cui A., Gong Y., Lu J., Liu X., Huo X. (2021). Development of Microsatellite Marker System to Determine the Genetic Diversity of Experimental Chicken, Duck, Goose, and Pigeon Populations. BioMed Res. Int..

[38] Cobo-Simón I., Márquez-Rodríguez J., Méndez-Cea B., Gallego F.J., Pérez-Fernández M. (2020). Understanding the morphological and genetic distinctiveness of the Spanish pouter pigeons: The Marchenero Pouter as a case study. Ibis.

[39] Dybus A., Kmieć M. (2002). PCR-RFLPs within the Iactate dehydrogenase (LDH-A) gene of the domestic pigeon (*Columba livia* var. *domestica*). J. Appl. Genet..

[40] Dybus A., Pijanka J., Cheng Y.H., Sheen F., Grzesiak W., Muszyńska M. (2006). Polymorphism within the LDHA gene in the homing and non-homing pigeons. J. Appl. Genet..

[41] Proskura W.S., Kustosz J., Dybus A., Lanckriet R. (2015). Polymorphism in dopamine receptor D4 gene is associated with pigeon racing performance. Anim. Genet..

[42] Jacob G., Prévot A.C., Baudry E. (2016). Feral Pigeons (*Columba livia*) Prefer Genetically Similar Mates despite Inbreeding Depression. PLoS ONE.

[43] de Groot M., van Haeringen W.A. (2017). An evaluation of the International Society for Animal Genetics recommended parentage and identification panel for the domestic pigeon (*Columba livia domestica*). Anim. Genet..

[44] Lee J.C.I., Tsai L.C., Kuan Y.Y., Chien W.H., Chang K.T., Wu C.H., Linacre A., Hsieh H.M. (2007). Racing pigeon identification using STR and chromo-helicase DNA binding gene markers. Electrophoresis.

[45] Giunchi D., Mucci N., Bigi D., Mengoni C., Baldaccini N.E. (2020). Feral pigeon populations: Their gene pool and links with local domestic breeds. Zoology.

[46] Gazda M.A., Andrade P., Afonso S., Dilyte J., Archer J.P., Lopes R.J., Faria R., Carneiro M. (2018). Signatures of selection on standing genetic variation underlie athletic and navigational performance in racing pigeons. Mol. Biol. Evol..

[47] Seki S.I. (2006). Application of molted feathers as noninvasive samples to studies on the genetic structure of pigeons (Aves: Columbidae). J. For. Res..

