# Supplementary material for: Genetic Structure of Racing Pigeons (Columba livia) Kept in Poland Based on Microsatellite Markers
Source: Genes (Basel). 2022 Jun 29;13(7):1175. doi: 10.3390/genes13071175 (PMC9318851; doi:10.3390/genes13071175)
Supplement: Supplementary file 1 [file genes-13-01175-s001.zip › genes-1747697-Supplementary.pdf]

genes

MDPI

Article

Genetic Structure of Racing Pigeons (*Columba livia*) Kept in Poland Based on Microsatellite Markers

Angelika Podbielska * and Anna Radko

Department of Animal Molecular Biology, National Research Institute of Animal Production, Krakowska 1, 32-083 Balice, Poland; anna.radko@iz.edu.pl
* Correspondence: angelika.podbielska@iz.edu.pl

Supplementary Materials

Table S1. The Evanno table output from STRUCTURE HARVESTER on a dataset containing 519 individuals.

K	Reps	Mean LnP(K)	Stdev LnP(K)	Ln'(K)	|Ln''(K)|	Delta K
1	10	-24287.320000	0.122927	—	—	—
2	10	-23826.230000	6.429109	461.090000	6.970000	1.084132
3	10	-23372.110000	20.831037	454.120000	59.920000	2.876477
4	10	-22977.910000	13.627868	394.200000	179.700000	13.186215
5	10	-22763.410000	72.231594	214.500000	115.200000	1.594870
6	10	-22433.710000	27.271860	329.700000	94.390000	3.461077
7	10	-22198.400000	22.630756	235.310000	44.120000	1.949559
8	10	-22007.210000	20.028505	191.190000	19.550000	0.976109
9	10	-21835.570000	46.606367	171.640000	54.640000	1.172372
10	10	-21718.570000	33.777575	117.000000	—	—

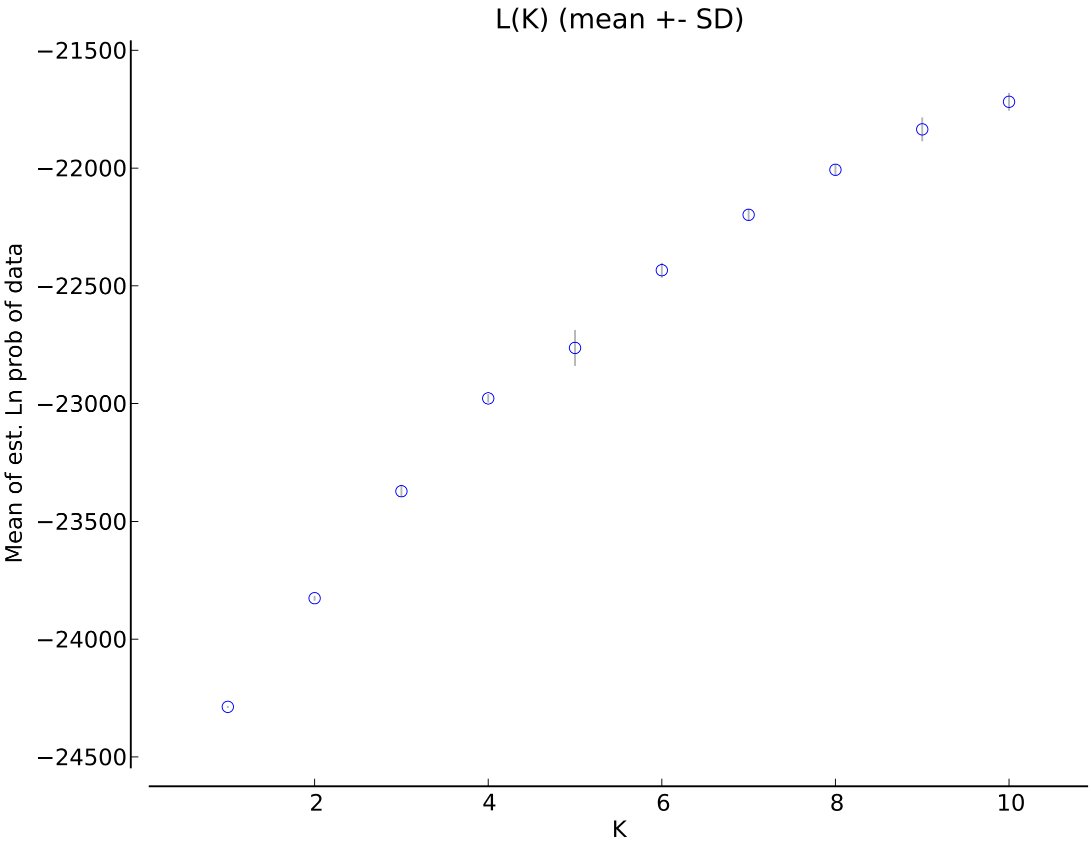

Figure S1. Plot of mean likelihood L(K) and variance per K value from STRUCTURE HARVESTER on a dataset containing 519 individuals.

Table S2. The Evanno table output from STRUCTURE HARVESTER on a dataset containing 519 individuals divided into five populations by country of origin.

K	Reps	Mean LnP(K)	Stdev LnP(K)	Ln'(K)	|Ln''(K)|	Delta K
1	10	-24287.400000	0.124722	—	—	—
2	10	-23825.260000	5.637415	462.140000	23.110000	4.099397
3	10	-23386.230000	52.843523	439.030000	25.060000	0.474230
4	10	-22972.260000	5.232633	413.970000	186.300000	35.603493
5	10	-22744.590000	52.633142	227.670000	65.330000	1.241233
6	10	-22451.590000	56.082250	293.000000	33.350000	0.594662
7	10	-22191.940000	11.797665	259.650000	86.480000	7.330264
8	10	-22018.770000	45.539191	173.170000	8.680000	0.190605
9	10	-21854.280000	47.616496	164.490000	32.730000	0.687367
10	10	-21722.520000	52.546356	131.760000	—	—

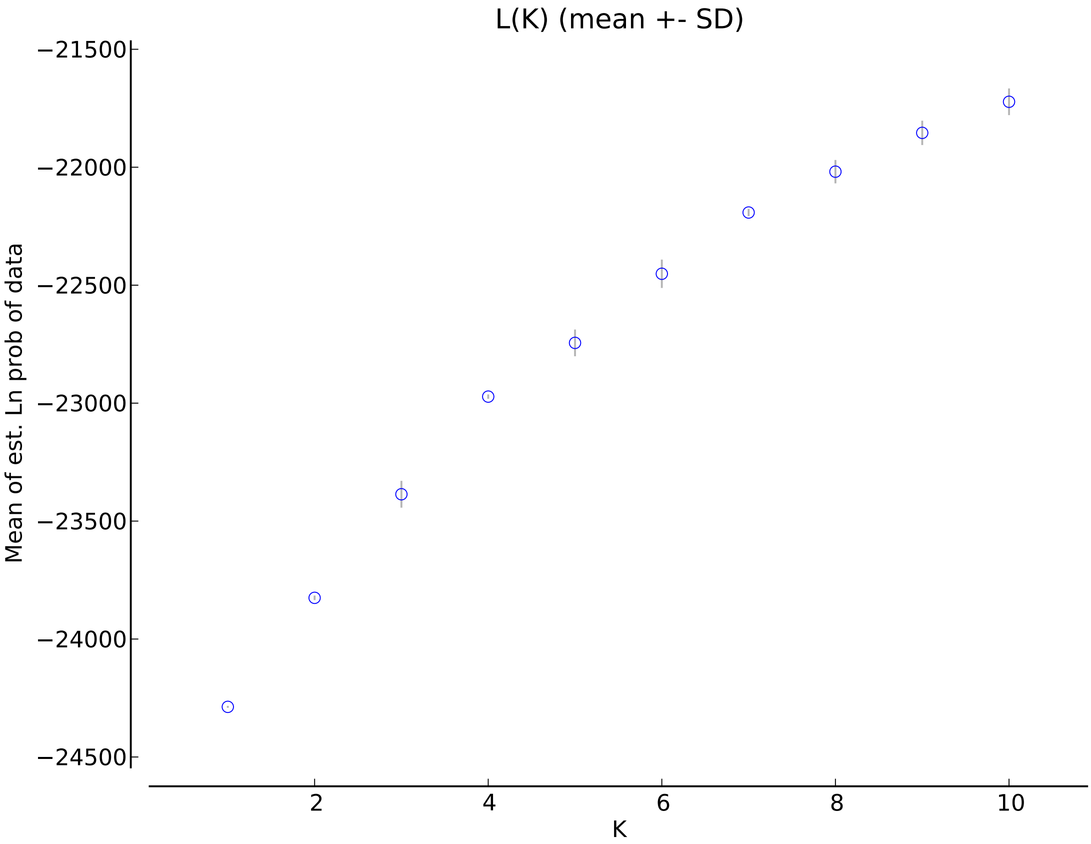

Figure S2. Plot of mean likelihood L(K) and variance per K value from STRUCTURE HARVESTER on a dataset containing 519 individuals divided into five populations by country of origin.

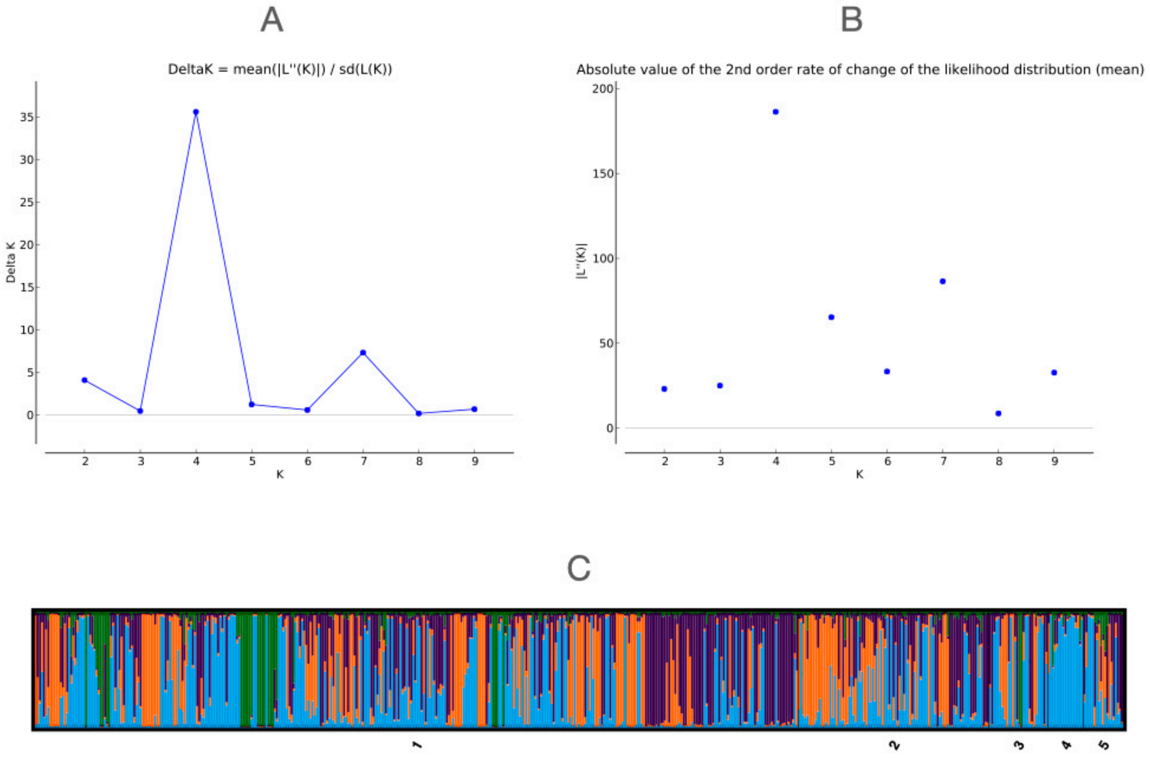

Figure S3. (A) Delta K values obtained with STRUCTURE HARVESTER. (B) Rate of change in the likelihood distribution (mean) obtained with STRUCTURE HARVESTER. (C) STRUCTURE software clustering at K=4, where: 1–PL; 2–BE; 3–DV; 4–SK; and 5–NL.

Table S3. Pairwise F_{ST} values, where: 1–PL; 2–BE; 3–DV; 4–SK; and 5–NL.

**1**	**2**	**3**	**4**	**5**	
0,000					1
0,003	0,000				2
0,006	0,007	0,000			3
0,031	0,034	0,037	0,000		4
0,010	0,013	0,014	0,036	0,000	5

Table S4. Pairwise population Nm values based on F_{ST} values, where: 1–PL; 2–BE; 3–DV; 4–SK; and 5–NL.

**1**	**2**	**3**	**4**	**5**	
0.000					1
140.825	0.000				2
114.017	72.601	0.000			3
5.644	5.179	4.967	0.000		4
87.262	40.269	62.816	5.907	0.000	5
